# Supplementary material for: Potential Therapeutic Effects of Bifidobacterium breve MCC1274 on Alzheimer’s Disease Pathologies in AppNL-G-F Mice
Source: Nutrients. 2024 Feb 15;16(4):538. doi: 10.3390/nu16040538 (PMC10893354; doi:10.3390/nu16040538)
Supplement: Supplementary file 1 [file nutrients-16-00538-s001.zip › nutrients-2864030-supplementary.pdf]

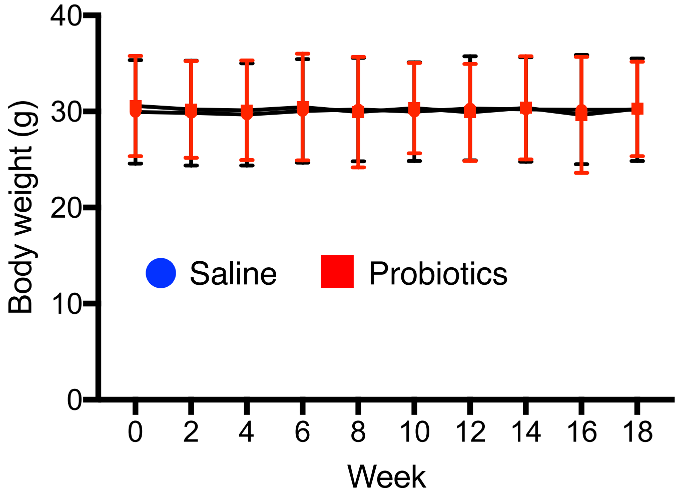


**Supplementary Figure S1:** Effect of oral *B. breve* MCC1274 supplementation on average body weight (g) in 17-month-old *App*$^{NL\text{-}G\text{-}F}$ mice. Body weight was monitored every 2 weeks over the 4 months of probiotic supplementation in *App*$^{NL\text{-}G\text{-}F}$ mice (n = 16-17 in each group). Data was analyzed by Student's t-test.
